# Suspected ocular toxocariasis and macular heterotopia

**Published:** 2020

**Authors:** Miriam Rahhal-Ortuño, Luis Alonso-Muñoz, Eduardo Fonseca-Fernández, MS Rahhal

**Affiliations:** * Department of Ophthalmology, Hospital Universitari i Politecnic La Fe, Valencia, Spain; ** Clínica Oftalmológica Rahhal, Valencia, Spain

**Keywords:** macular heterotopia, granuloma, ocular toxocariasis

## Abstract

We report a case of an asymptomatic Caucasian male who attended our clinic for a routine check-up and macular heterotopia associated with lesions compatible with ocular toxocariasis were found.

## Introduction

Toxocariasis is a zoonotic disease, which can be asymptomatic in humans or cause visceral, ocular, or neurological manifestations, the retina and vitreous humor being the most affected ocular tissues. Toxocara larvae generally access the human organism through the intestines and from there they are able to migrate to different organs [**1**]. Infection is more frequent in children; soil ingestion, exposure to dogs and pica, being described as the most important risk factors [**2**]. 

## Case presentation

**Fig. 1:**
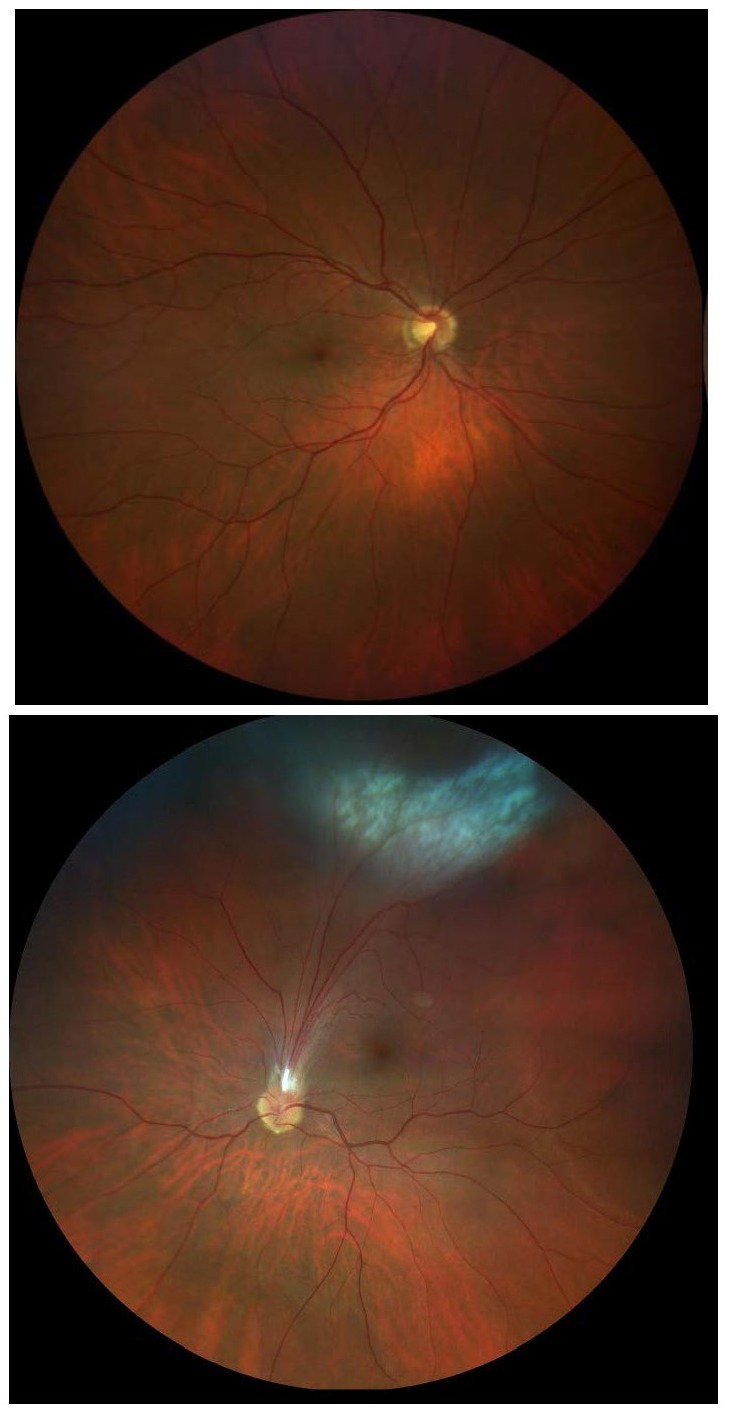
Right eye’s fundus examination with no significant findings. Left eye’s fundus examination shows a whitish peripheral granuloma associated to retinal folds extending towards the papilla and macular heterotopia

A 55-year-old Caucasian male attended our clinic for a routine ophthalmological check-up. Visual acuity was 1/ 1 using Snellen’s visual acuity chart. Anterior slit lamp examination and intraocular pressure were unaltered in both eyes. Right eye’s fundus examination did not reveal significant findings, but left eye’s fundus examination showed a superior peripheral granulomatous whitish hazy lesion with undefined limits, associated with retinal folds extending from the optic nerve head. Macular heterotopia was present as a result of force tractions caused by the folds (**Fig. 1**).

**Fig. 2:**
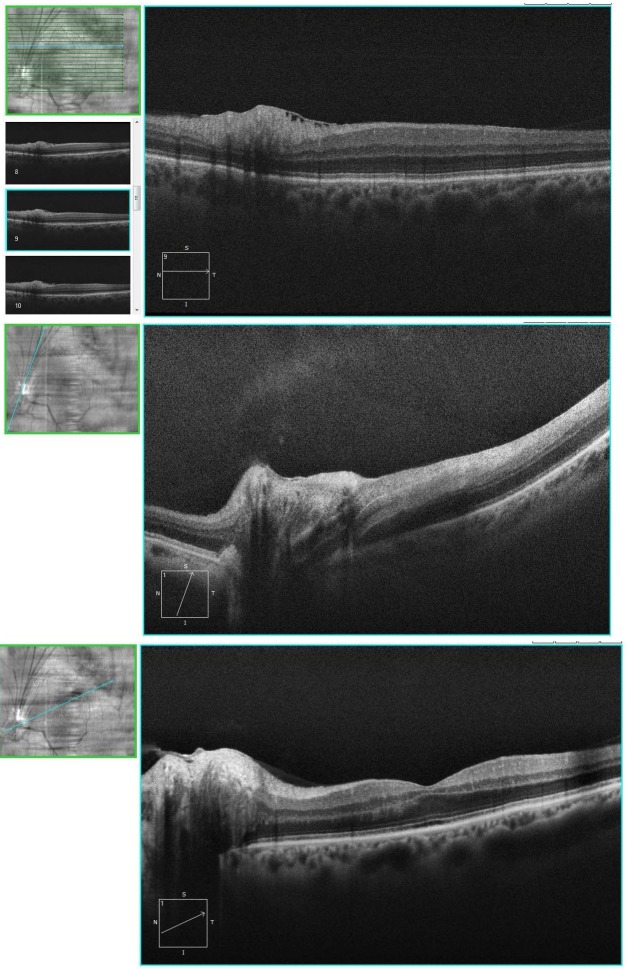
Left eye’s OCT imaging shows hyperreflectivity of internal layers in the affected area and distortion the optic nerve head contour, with no evident macular distortions

**Fig. 3:**
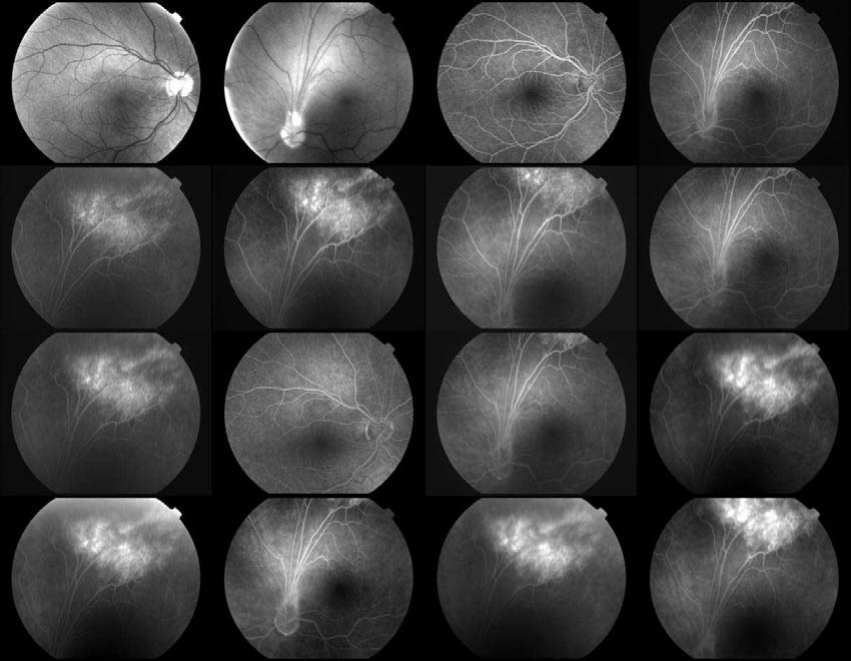
Fluorescein angiography showing hyperfluorescence of peripheral superior granuloma in the left eye

OCT images showed hyperreflectivity of internal layers regarding the affected area, as well as an epiretinal fold, which distorted the optic nerve head contour, with no evident macular distortions (**Fig. 2**). Fluorescein angiography was carried out showing evident hyperfluorescence in the left eye’s superior peripheral retina, especially during late phases (**Fig. 3**).

These findings, added to the fact that the patient’s medical history was unremarkable and extensive laboratory workup and chest radiograph did not reveal any significant results, strongly suggested toxocariasis as the cause of fundus alterations. Serologic testing was unrevealing.

As the patient remained asymptomatic, we decided not to carry out invasive diagnostic procedures.

## Results

By putting together the building blocks of this case - an asymptomatic patient with an incidental retinal finding compatible with a toxocariasis lesion and unremarkable laboratory workup and imaging - a presumptive diagnosis could be made.

Complete ophthalmological examination and high-quality imaging allowed us to document an infrequent case of ocular toxocariasis in order to make ophthalmologists more aware of this disease.

## Discussion

Ocular toxocariasis is uncommon and usually unilateral [**2**]. Its most common presentation has been found to be the presence of retinal granulomas, which may associate fibrosis, retinal distortions and tractional detachments causing vision loss and strabismus [**3**]. Other ocular manifestations that can be found are keratitis, hypopyon, vitreous abscess, and papillitis [**1**]. 

When there is no evident ocular inflammation, the presence of leukocoria is one of the most common signs for which patients may ask for a consultation. Differential diagnosis has to be done with retinoblastoma, retinopathy of prematurity, Coat’s disease and infectious endophthalmitis amongst others [**4**]. 

Definite diagnosis of ocular toxocariasis remains a challenge as it is only possible by histological demonstration and eye biopsies are rarely justified if patients are asymptomatic [**5**]. In addition, antibody levels in serum are very low or undetectable in a significant number of cases, diagnosis being essentially clinical [**1**,**4**,**5**]. In the vast number of cases, the diagnosis of ocular toxocariasis remains only presumptive [**4**]. 

## Conclusion

Although ocular toxocariasis is an infrequent disease, it is important for ophthalmologists to be aware of the characteristic lesions it causes, so that a correct diagnostic approach could be made. An important fact that ophthalmologists should also take into account is that serological tests are unremarkable in a significant number of patients, and this should not rule out the presence of toxocariasis. 

**Conflict of interest**

Authors declare no conflict of interest.

**Acknowledgements**

There are no funders to report for this submission.

**Informed consent**

Consent was gathered from the patient in order to obtain and publish these images.
